# Suppressor of Cytokine Signaling (SOCS) Genes Are Silenced by DNA Hypermethylation and Histone Deacetylation and Regulate Response to Radiotherapy in Cervical Cancer Cells

**DOI:** 10.1371/journal.pone.0123133

**Published:** 2015-04-07

**Authors:** Moon-Hong Kim, Moon-Sun Kim, Wonwoo Kim, Mi Ae Kang, Nicholas A. Cacalano, Soon-Beom Kang, Young-Joo Shin, Jae-Hoon Jeong

**Affiliations:** 1 Department of Obstetrics and Gynecology, Korea Cancer Center Hospital, Korea Institute of Radiological and Medical Sciences, Seoul, Korea; 2 Research Center for Radiotherapy, Korea Institute of Radiological and Medical Sciences, Seoul, Korea; 3 Department of Radiation Oncology, University of California Los Angeles, Los Angeles, California, United States of America; 4 Department of Obstetrics and Gynecology, Konkuk University Medical Center, Seoul, Korea; 5 Department of Radiation Oncology, Inje University Sanggye Paik Hospital, Seoul, Korea; University of Newcastle, AUSTRALIA

## Abstract

Suppressor of cytokine signaling (SOCS) family is an important negative regulator of cytokine signaling and deregulation of SOCS has been involved in many types of cancer. All cervical cancer cell lines tested showed lower expression of SOCS1, SOCS3, and SOCS5 than normal tissue or cell lines. The immunohistochemistry result for SOCS proteins in human cervical tissue also confirmed that normal tissue expressed higher level of SOCS proteins than neighboring tumor. Similar to the regulation of SOCS in other types of cancer, DNA methylation contributed to SOCS1 downregulation in CaSki, ME-180, and HeLa cells. However, the expression of SOCS3 or SOCS5 was not recovered by the inhibition of DNA methylation. Histone deacetylation may be another regulatory mechanism involved in SOCS1 and SOCS3 expression, however, SOCS5 expression was neither affected by DNA methylation nor histone deacetylation. Ectopic expression of SOCS1 or SOCS3 conferred radioresistance to HeLa cells, which implied SOCS signaling regulates the response to radiation in cervical cancer. In this study, we have shown that SOCS expression repressed by, in part, epigenetically and altered SOCS1 and SOCS3 expression could contribute to the radiosensitive phenotype in cervical cancer.

## Introduction

Members of the suppressor of cytokine signaling (SOCS) family of proteins play key roles in the negative regulation of cytokine signal transduction. These proteins act in a negative feedback loop, inhibiting the cytokine-activated Janus kinase/signal transducers and activators of transcription (JAK/STAT) signaling pathway to modulate cellular responses [1]. SOCS1 appears to have tumor suppressor activity [2] and restoration of SOCS1 gene expression causes growth suppression and induction of apoptosis in HCC cells [3]. Recently, Sobti et al showed loss of SOCS1 expression through promoter methylation in more than 60% of cervical cancer cases and proposed the importance of SOCS1 downregulation in HPV-induced cervical carcinogenesis [4]. SOCS3 is involved in the development and progression of several malignancies, and there are indications that SOCS3 has different functions depending on the tumor origin. In human lung [5], hepatocellular [6], and head and neck cancer [7], SOCS3 is silenced by hypermethylation, which gives a growth advantage to cancer cells. In contrast, SOCS3 is detectable in breast cancer [8], and SOCS3 expression is increased during the development and progression of prostate cancer [9].

It is generally accepted that cervical cancers are radiosensitive and treatment outcomes are still promising even after the tumor is diagnosed too late for treatment using radical hysterectomy. In Korea, cervical cancer is a major health concern for women, accounting for 9.8% of new female cancer cases. Although incidence and mortality rates have been decreasing, the incidence of cervical cancer in the elderly is increasing [10]. However, most of the treatment failures in advanced cervical cancersoriginate from the development of radioresistance, a problem that we have not yet overcome. Interestingly, SOCS1 sensitizes glioblastoma cells to radiation, whereas SOCS3 enhances tumor cell survival and radioresistance [11]. Zhou et al. suggested that targeting SOCS expression or function in glioblastoma cells may be a useful strategy to sensitize tumor cells to ionizing radiation. While the DNA damage response pathway is critical for managing genotoxic stress, the outcome of the response is highly dependent on the cellular context. Clearly, other signaling pathways activated in the cell at the time of DNA damage can modulate the response and alter the output of DNA damage-induced signal transduction [12].

In the present study, we have analyzed SOCS1, SOCS3, and SOCS5 gene expression in a panel of cell lines representing primary, *de novo* human cervical cancersthat are radiosensitive. We have shown that SOCS1, SOCS3, and SOCS5 expression is repressed, in part, epigenetically by DNA hypermethylation and histone deacetylation. We show that altered SOCS1 and SOCS3 expression may contribute to the radiosensitive phenotype in cervical cancer.

## Materials and Methods

### Cell culture, normal cervix tissue and inhibitor treatment

The human cervical carcinoma cell lines CaSki, HeLa, ME-180, and SiHa were obtained from the Korean Cell Line Bank (KCLB, Seoul, Korea). Normal human fibroblast cell lines CCD-18Lu, CCD-18Co, and WI-38 were purchased from the American type culture collection (ATCC, Manassas, VA, USA). The CaSki and ME-180 cell lines were maintained in RPMI-1640 (PAA Laboratories, Pasching, Austria) and the HeLa, SiHa, and normal fibroblast cell lines were maintained in DMEM (PAA Laboratories), supplemented with 10% fetal bovine serum (Lonza, Walkersville, MD, USA) and 100 units of penicillin and streptomycin (PAA Laboratories). All cells were cultured in a humidified incubator with 5% CO_2_ at 37°C. For inhibition of DNA methyltransferase, cells were seeded at a density of 2–5 × 10^5^/100-mm dishes and treated with 5-azacytidine (5-AzaC) at 10 μM for 72 h. Media and 5-AzaC were replaced every 24 h. For inhibition of histone deacetylase, cells were treated with Trichostatin A (TSA) at 100 or 200 nM for 16 h. Both inhibitors were purchased from Sigma Chemical Co. (St. Louis, MI, USA). A normal cervix tissue was obtained from a patient who underwent hysterectomy for myoma uteri. We obtained a written informed consent from a patient before surgery. The Institutional Review Board approved the consent procedure and the study protocol (K-1103-003-016) at the Korea Institute of Radiological and Medical Science.

### RealtimeqRT-PCR

Total RNA from cells and tissue was isolated using the Hybrid-R Total RNA Purification Kit (GeneAll, Seoul, Korea). A total of 1 μg of total RNA was reverse transcribed using the PrimeScript RT Reagent Kit (Takara Bio Inc, Japan) with 25 pmol of oligo-dT primer and 50 pmol of random hexamer. cDNA was diluted 1/10 with EZ Dilution Buffer (Takara Bio Inc), and 2 μL was used as template in a 20 μL PCR reaction. Quantitative PCR was performed using Premix Ex Taq (Takara Bio Inc) in a CFX-96 thermocycler (Bio-Rad Laboratories, Hercules, CA, USA). The following primers were used for PCR: Cyclophilin A (CypA) (sense, ACG GCG AGC CCT TGG; antisense, TTT CTG CTG TCT TTG GGA CCT), SOCS1 (sense, TTT TCG CCC TTA GCG TGA A; antisense, CAT CCA GGT GAA AGC GGC), SOCS3 (sense, GCT CCA AAA GCG AGT ACC AGC; antisense, AGT AGA ATC CGC TCT CCT GCA G), and SOCS5 (sense, AAA AGT TGG ATC TGT TCC TAT GCC; antisense, ACT ATT ATC TGA CTT GTT TG). Fluorogenic probes (6-carboxyfluorescein) were: CypA, CGC GTC TCC TTT GAG CTG TTT GCA; SOCS1, CCT CGG GAC CCA CGA GCA TCC; SOCS3, TTG CGC ACG GCG TTC ACC AC; SOCS5, AGG ATG ATT TTG TGA ACC GTG AAG TAC GTG A. DNMT1 gene expression was measured using SYBR premix Ex Taq II kit (Takara Bio Inc) with primers (sense, CTT CTT CAG CAC AAC CGT CA; antisense, GAA GAG CCG GTA GGT GTC AG). The relative quantification of gene expression was calculated with the ddC(t) method, where CypA mRNA was used for normalization. The PCR program was as follows: after an initial pre-incubation step at 95°C for 3 min, there were 45 cycles, each consisting of 95°C for 15 s and 60°C for 60 s. The last amplification cycle was followed by a melt curve analysis to make sure about the specificity of the qRT-PCR.

### Immunohistochemistry

All tissue samples were obtained with full ethical approval from the KIRAMS institutional review board (IRB No. K-1103-003-016). Immunohistochemical staining for SOCS1, SOCS3, and SOCS5 were performed by an automated immunohistochemical stainer (Autostainer 480, Thermo Fisher Scientific, Fremont, CA, USA) on formalin-fixed paraffin embedded tissue sections of squamous cell carcinomas obtained from uterine cervix. Sections were deparaffinized with xylene for 15 min and pretreated in a microwave oven using 0.01 M citrate buffer (pH 6.0) for 30 min. Sections were incubated with primary antibodies directed against SOCS1 (1:25, ab62584, Abcam, Cambridge, UK), SOCS3 (1:100, ab16030, Abcam) and SOCS5 (1:100, sc-100858, Santa Cruz Biotechnology, Santa Cruz, CA, USA) for 60 min at room temperature and detected using UltraVision LP Detection System HRP Polymer & DAB Plus Chromogen (Thermo Fisher Scientific).

### Methylation-specific PCR (MSP)

MSP was carried out to investigate the methylation status of the promoter regions of the SOCS1, SOCS3, and SOCS5 genes. After bisulfite treatment to modify the DNA, PCR was performed to distinguish methylated from unmethylated DNA as described by Herman et al [13]. Genomic DNA was extracted from cell lines using Exgene Cell SV Kit (GeneAll, Korea) and bisulfite modification of genomic DNA was carried out using the EZ DNA methylation Kit (Zymo Research, CA, USA) according to the manufacturer's instructions. The bisulfite-treated DNA was amplified with either a methylation-specific primers or primers specific to the unmethylated sequence. Briefly, a 20 μL reaction volume containing 25 ng bisulfite modified DNA, 1× PCR buffer, 1.5 mM MgCl_2_, 0.25 mM dNTPs, 0.5 μM specific primer mix and 1 unit Ex-Taq Hot Start enzyme (Takara BioInc) was used. The PCR products were electrophoresed on a 2% agarose gel, stained with RedSafe Nucleic Acid Staining Solution (Intron, Korea) and visualized under UV illumination. Primer lists and PCR conditions for MSP are summarized in S1 Table.

### Bisulfite sequencing

Bisulfite-treated genomic DNA was amplified by the primers listed in S2 Table. The PCR products were cloned into the Topo TA cloning kit (Invitrogen, Carlsbad, CA, USA) or the T-blunt PCR cloning Kit (Solgent, Daejeon, KOREA). Five randomly picked clones were sequenced and aligned using QUMA (Quantification tool for Methylation Analysis).

### Gene silencing by shRNA

The lentiviral shRNA expression plasmids targeting DNMT1 were generated by cloning oligonucleotides into the pLKO.1 puro vector. The sequences of hairpins targeting DNMT1 are: GCCGAATACATTCTGATGGATCTCGAGATCCATCAGAATGTATTCGGC (TRCN0000021890) and GCCCAATGAGACTGACATCAACTCGAGTTGATGTCAGTCTCATTGGGC (TRCN0000021891). The control scrambled shRNA expressing plasmid was obtained from Addgene with the following hairpin sequence: CCTAAGGTTAAGTCGCCCTCGCTCGAGCGAGGGCGACTTAACCTTAGG. For packaging, pCMV-dR8.2 dvpr and pCMV-VSVG were used, both of which were purchased from Addgene (Cambridge, MA, USA). The production of viral particles and transduction of target cells was conducted following the pLKO.1 protocol from Addgene. Supernatants from 293T cells transfected by the shRNA and packaging vectors were collected 48 and 72 h post transfection followed by concentration of the virus. Concentrated virus was used for infections in the presence of 8 μg/mL polybrene. One day after infection, puromycin (1 μg/mL) was applied for 4 days to select transduced cells, which were harvested for realtime PCR analysis.

### Cloning of SOCS overexpressing retroviral constructs

Constructs for overexpressing SOCS1, SOCS3, and SOCS5 were made by cloning the SOCS1, SOCS3, and SOCS5 ORF into the pLNCX2 retroviral vector (Clontech). The SOCS1 and SOCS3 genes of mouse origin were kindly provided from Dr. Cacalano NA (UCLA, CA). The *Bam*HI fragment containing SOCS1 cDNA tagged with Myc at C-terminus or SOCS3 cDNA tagged with FLAG at C-terminus was subcloned into *Bgl*II site of pLNCX2. Clones with correct orientation were screened with *Eco*RI digestion and then confirmed by sequencing. The SOCS5 gene of mouse origin was purchased from Openbiosystems (MMM1013-9202191) and amplified with sense (5’-AAACTCAGATCTACCATGGATGGATAAAGTGGGGAAAATG-3’) and antisense(5’-AGATATGGATCCCTCGAGCTACTACTTATCGTCATCATCTTTATAATCGATCTTTGCCTTGACTGGTTCTC-3’) primers. A *Bgl*II and *Sal*I fragment containing SOCS5 with FLAG at C-terminus was subcloned into the *Bgl*II and *Xho*I site of pLNCX2 retroviral vector.

### Virus production and establishing stable cells

SOCS overexpressing retroviral vectors and packaging plasmids, pCMV-Gag-Pol and pCMV-VSVG, were cotransfected into 293T cell. Supernatants from 293T cells were collected 48 and 72 h post transfection followed by concentration of the retrovirus and used to infect HeLa cells in the presence of 8 μg/mL polybrene. One day after infection, G418 (200 ng/mL) was applied until stable colonies formed. The colonies were pooled to check the SOCS overexpression and used for clonogenic assay.

### Western blot analysis

Cells were lysed with SDS sample buffer (125 mM Tris-HCl, pH 6.8, 4% SDS, 15% glycerol, and 0.004% bromophenol blue) and then boiled for 10 min. Protein content was measured with the BCA Protein Assay Reagent (Intron). Equal amounts of cell lysate were separated by SDS-polyacrylamide gels, transferred to a nitrocellulose membrane, immunoblotted and detected by chemiluminescence using ECL detection reagents. Anti-myc, anti-actin and horseradish peroxidase-conjugated secondary antibodies were purchased from Santa Cruz Biotech (Santa Cruz, CA, USA) and the anti-FLAG antibody was from Sigma Chemical Co.

### Clonogenic assay

Clonogenic survival was defined as the ability of cells to maintain clonogenic capacity and to form colonies. Briefly, cells were harvested from exponential phase cultures by trypsinization, counted and seeded for colony formation. On the following day, gamma radiation was delivered using a dual-source ^137^Cs unit at a dose rate of 3.2 Gy/min with a GC-3000 Elan irradiator (MDS Nordion, Ontario, Canada). After 14 days, colonies were stained with 0.4% crystal violet (Sigma Chemical Co.). The colonies were counted and groups of >30 cells were considered as colonies. The plating efficiency (PE) represents the percentage of cells seeded that grow into colonies under a specific culture condition for a given cell line. The survival fraction, expressed as a function of irradiation, was calculated as follows: Survival fraction = colonies counted/(cells seeded × PE/100). We repeated the experiments five times to ensure reproducibility.

### Statistical analysis

Data are given as mean ± SD. The paired Student’s *t*-test was performed and *p*-values less than 0.05 were considered statistically significant.

## Results

### SOCS expression profile incervical cancer cells

**Fig 1:**
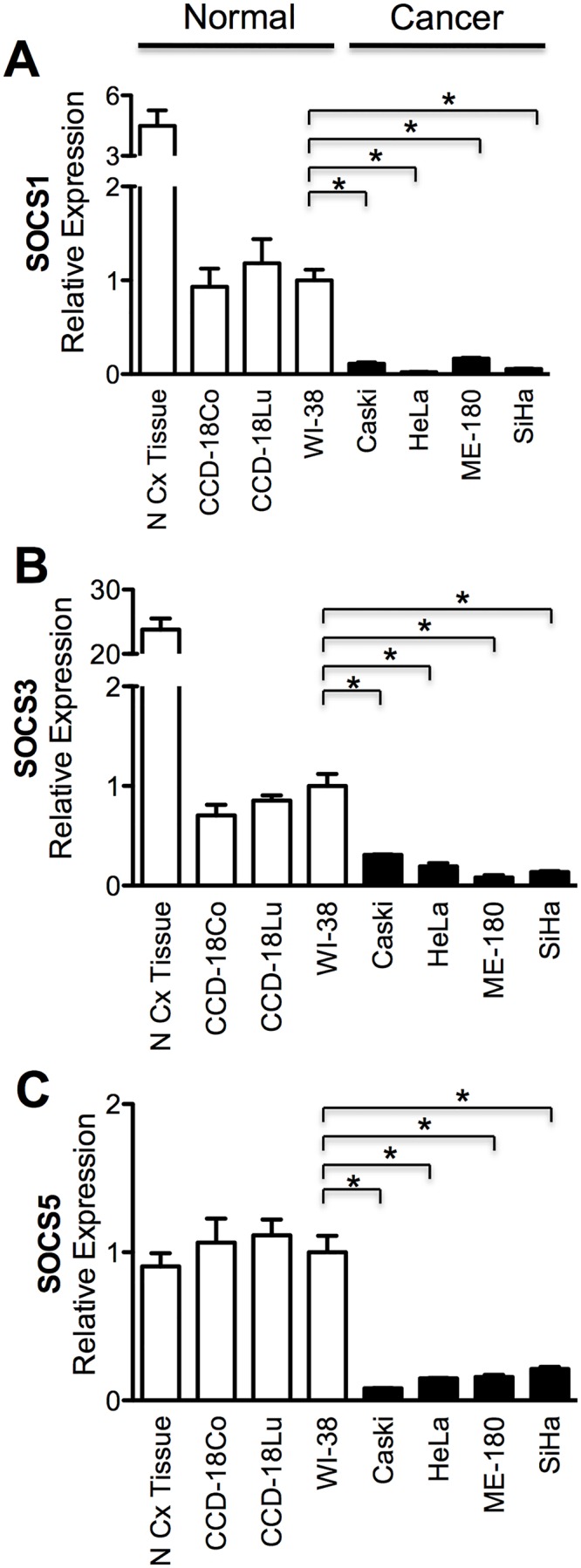
SOCS genes were downregulated in cervix cancer. Expression of SOCS1 (A), SOCS3 (B), and SOCS5 (C) gene were examined by qRT-PCR in normal cervix (N Cx) tissue, three normal fibroblast cell lines (CCD-18Co, CCD-18Lu, WI-38) and cervix cancer cell lines (CaSki, HeLa, ME-180, SiHa). Expression level of normal control samples marked as open bar and cervix cancer cell closed bar. Asterisk (*), statistically significant (*p* <0.05).

**Fig 2:**
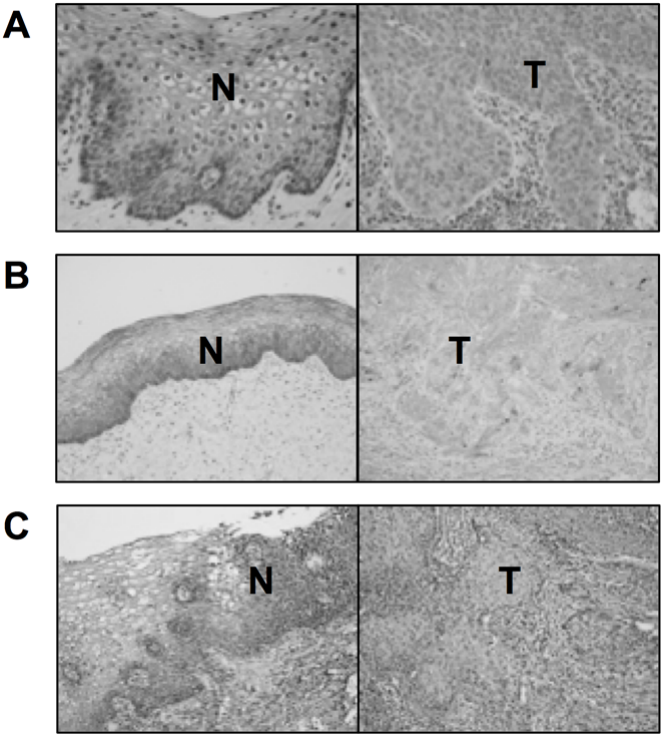
Immunohistochemical analysis of SOCS expression in human cervix cancer and its normal surrounding tissue. Expression level of SOCS1 (A), SOCS3 (B), and SOCS5 (C) in tumor (T) and neighboring normal (N) epithelial cell of a surgically removed patient sample were compared.

To reveal the possible role of SOCS in cervical cancer development and proliferation, expression of SOCS1, SOCS3,and SOCS5 was examined in several human cervical cancer cell lines, CaSki, HeLa, ME-180, and SiHa. The mRNA level of SOCS1 (Fig 1A), SOCS3 (Fig 1B), and SOCS5 (Fig 1C) was significantly lower in all cervical cancer cells tested than in normal cervix tissue and established normal colon (CCD-18Co) or lung fibroblast (CCD-18Lu, WI-38) cell lines,

To confirm that the lower expression of SOCS1, SOCS3, and SOCS5 in cervical cancer cells is a general phenomenon observed in human tumors, immunohistochemical staining of SOCS proteins was performed in human cervical cancer tissue and in the surrounding normal tissue as a control (Fig 2). All three SOCS proteins were stained darker in normal (N) tissue compared to the tumor (T) area. All these results led to the conclusion that SOCS1, SOCS3, and SOCS5 expression ishigher in normal tissue than in cervical cancer, and support the possibility that downregulation of SOCS might contribute to the tumorigenesis or maintenance of cervical cancer.

### DNA methylation analysis of SOCS gene promoter

**Fig 3:**
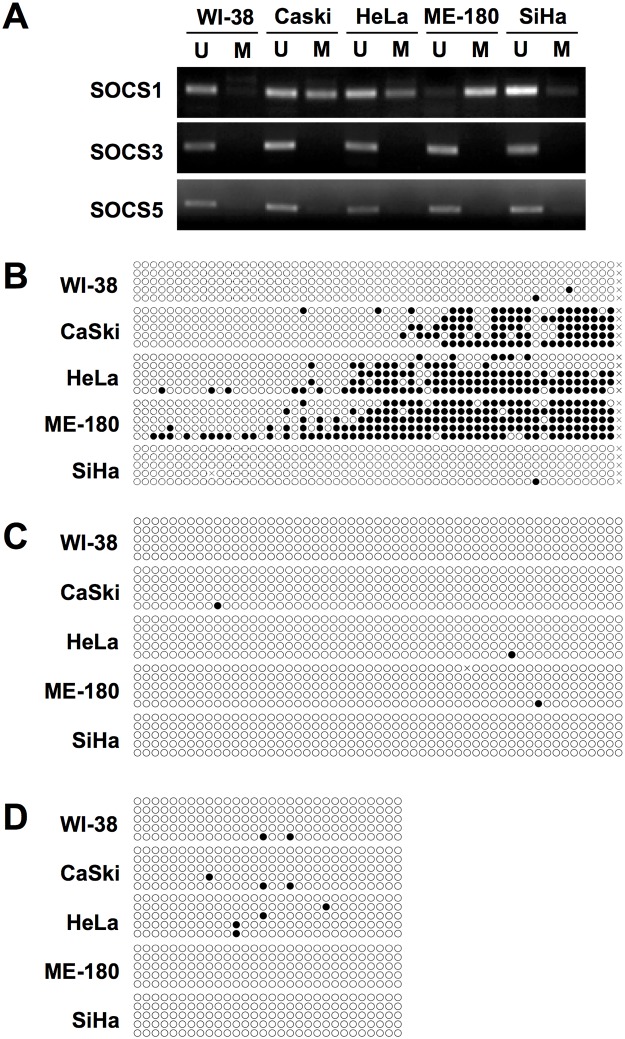
DNA methylation analysis of SOCS gene promoter. (A) Methyl-specific PCR analysis. Bisulfite-treated genomic DNA was amplified with unmethylated (U) or methylated (M) DNA specific primers. (B-D) Bisulfite sequencing of SOCS1 (B), SOCS3 (C), and SOCS5 (D). Unmethylated CpG site in amplified promoter region was showed as an open circle and methylated CpG as a closed circle.

To determine whether DNA methylation contributes to the downregulation of SOCS proteins in cervical cancer, a methylation-specific PCR (MSP) assay was performed. SOCS1 expression was correlated with differential DNA methylation only in CaSki, HeLa, and ME-180 cells (Fig 3A). The SOCS1 promoter in SiHa cell was not methylated in this region, although SOCS1 transcription was downregulated in SiHa cells to a similar levelas other cervix cell lines. In contrast, no DNA methylation was detected in the SOCS3 and SOCS5 promoter region, at least in the CpG-enriched region examined in this experiment, of all cervix cancer cell lines (Fig 3A).

To confirm the MSP result, the promoter regions of SOCS1, SOCS3, and SOCS5 genes encompassing CpG islands were cloned and five independent clones were sequenced (Fig 3B–3D). Unmethylated CpG sites are indicated by an open circle and methylated CpG sites are indicated by a filled circle. DNA methylation was detected in the SOCS1 gene promoter in CaSki, HeLa and ME-180 cells but not in SiHa (Fig 3B), matching the MSP result (Fig 3A). In CaSki and HeLa, the methylated region of SOCS1 nearly equals the unmethylated region, while in ME-180 cells most of promoter region was methylated. Methylated CpG sites were not detected in the SOCS3 and SOCS5 promoters in cervical cancer cellsor normal cells (Fig 3C and 3D), coinciding with the MSP experiment (Fig 3A).

### Inhibition of DNA hypermethylation restores SOCS1 expression

**Fig 4:**
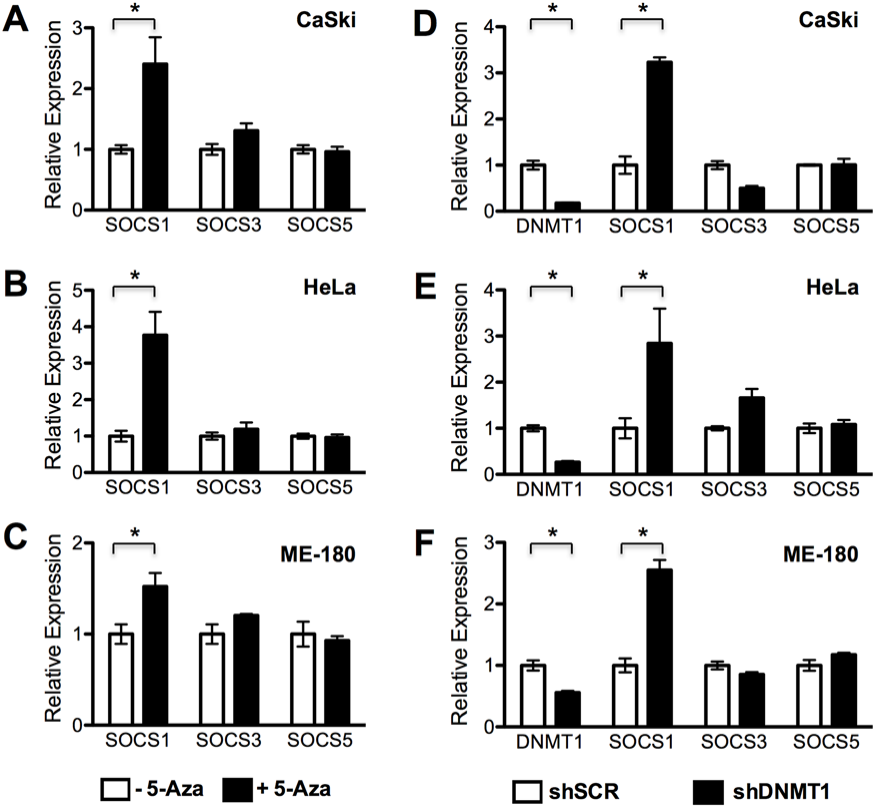
Effect of DNA methylation inhibitor and DNMT1 gene knockdown on SOCS gene expression. CaSki (A,D), HeLa (B,E), and ME-180 (C,F) cells were treated with 10 μM of 5-Azacytosine (5-Aza) for 72 h (A-C) or infected with control lentivirus (shSCR) or lentivirus expressing shRNA targeting DNMT1 (shDNMT1) (D-F). Knock-down of DNMT1 and SOCS gene expression was examined with qRT-PCR. Asterisk (*), statistically significant (*p* <0.05).

To determine whether DNA methylation of the SOCS1 promoter contributes to downregulation of SOCS1 gene expression *in vivo*,treatment with 5-AzaC was used to inhibit DNMT1 (Fig 4). In CaSki (Fig 4A) and HeLa (Fig 4B), SOCS1expression was prominently increased after 5-AzaC treatment. SOCS1 expression in ME-180 was slightly increased by treatment with the DNMT1 inhibitor (Fig 4C), possibly due tothe higher methylation of SOCS1 gene promoter in ME-180 cells (Fig 3A and 3B) than in the other cell lines. SOCS3 and SOCS5 expression was not affected by 5-AzaC treatment, supporting the previous result (Fig 3) that DNA methylation is not involved in the downregulation of SOCS3 and SOCS5. This result was confirmed by shRNA-mediated knock-downof DNMT1 (Fig 4D–4F). RNA interference (RNAi) is the pathway by which short interfering RNA (siRNA) or short hairpin RNA (shRNA) are used to inactivate the expression of target genes [14]. SOCS1 expression was increased by inhibition of DNMT1 while SOCS3 and SOCS5 expression increased little. These findings, along with MSP and bisulfite sequencing results, suggest a possibility that regulatory mechanisms other than hypermethylationare important for controlling SOCS expression.

### Histone acetylation regulates SOCS1 and SOCS3 gene expression

**Fig 5:**
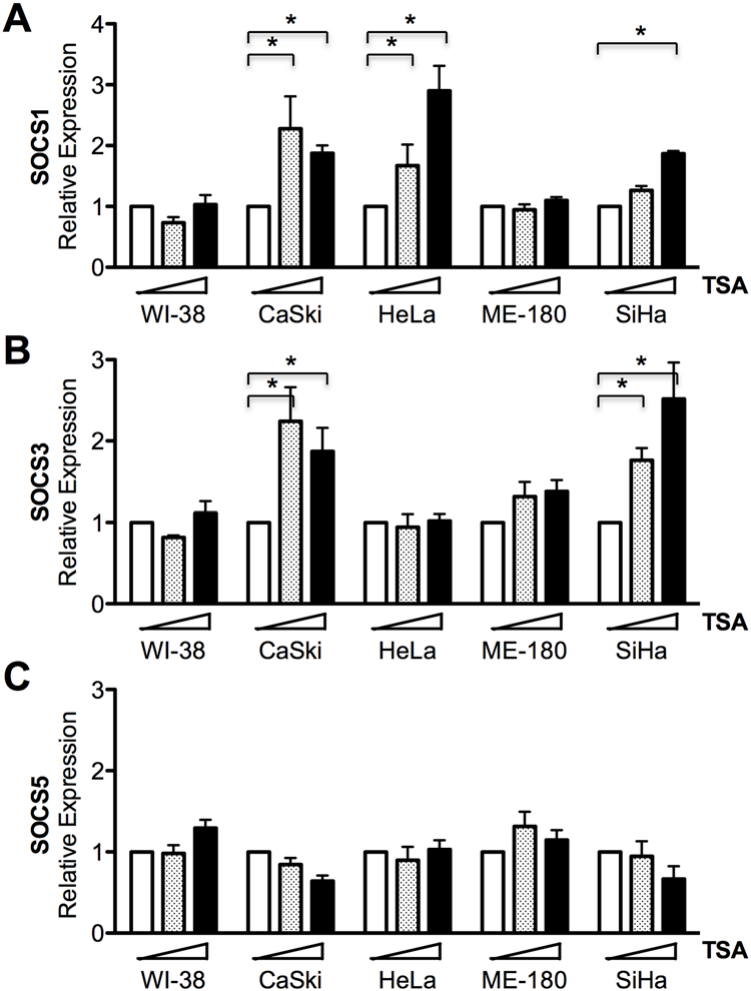
Effect of histone deacetylase inhibitor on SOCS gene expression. Normal lung fibroblast cells (WI-38) and cervix cancer cells were treated with 0, 100, or 200 nM of TSA for 16 h and SOCS1 (A), SOCS3 (B), and SOCS5 (C) gene expression was measured with qRT-PCR. Asterisk (*), statistically significant (*p* <0.05).

Histone deacetylation is a well-known epigenetic regulation mechanism that we tested for an association with the downregulation of SOCS gene expression. Treatment of Trichostatin A (TSA), a selective inhibitor of the class I and II mammalian histone deacetylase (HDAC) families of enzymes, increased SOCS1 gene expression in CaSki, HeLa, and SiHa but not ME-180 cells (Fig 5A). SOCS3 gene expression was also recovered in CaSki and SiHa cells after TSA treatment (Fig 5B). However, the expression of these genes was not affected by TSA in WI-38, a normal control lung fibroblast cell line. These results suggest that SOCS1 and SOCS3 are downregulated by HDAC in several cervix cancer cells. This is the first report that SOCS expression is affected by not only hypermethylation but also histone deacetylation in solid tumor cells. In contrast, SOCS5 gene expression was not changed at all by HDAC inhibition in WI-38 or in any of the cervical cancer cells tested (Fig 5C). Further study is required to characterize the mechanisms suppressing SOCS gene expression in cervical cancer.

### Overexpression of SOCS1 or SOCS3 endow radioresistance to HeLa cells

**Fig 6:**
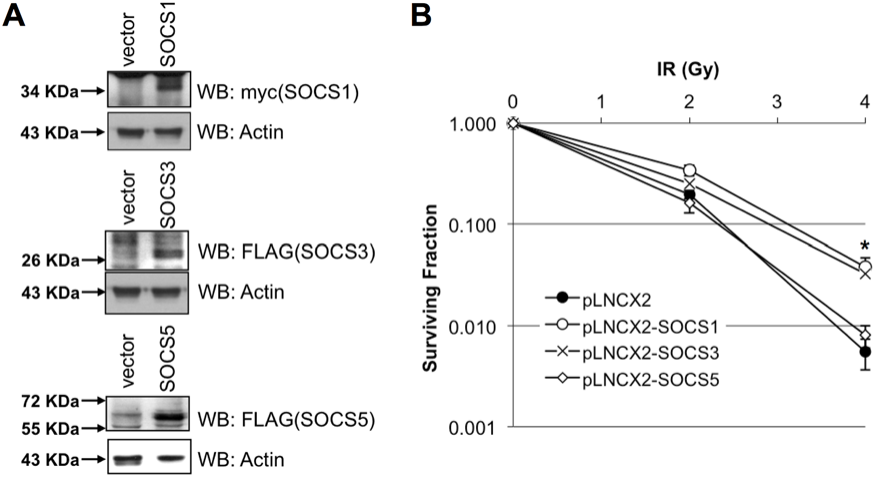
Overexpression of SOCS1 or SOCS3 increased radioresistance of HeLa cells. SOCS1, SOCS3, or SOCS5 overexpressing HeLa cells were established using pLNCX2 retroviral vector and overexpression was confirmed by western blot analysis (A). A clonogenic assay was performed after exposure to the indicated dose of radiation in SOCS-overexpressing cells (B). Values are means ± standard deviation for triplicate wells for each dose point. Asterisk (*), statistically significant (*p* <0.05)

To examine the biological effects of increased SOCS gene expression in cervical cancer cells, several HeLa cells were established by infection with retroviruses overexpressing the SOCS gene. Expression of the exogenous SOCS gene in neomycin resistant HeLa cells was confirmed by western blotting (Fig 6A). There was no significant difference observed in cell proliferation or viability between mock or SOCS-overexpressing HeLa cells (data not shown). As SOCS1 and SOCS3 have been involved in response to radiation in glioblastoma [11], clonogenic assays were performed to evaluate the possible contribution of SOCS gene expression to radioresponsivity. As shown in Fig 6, overexpression of SOCS1 or SOCS3 make HeLa cells more resistant to radiation than control cells (Fig 6B). However, the radiosensitivity of HeLa cells overexpressing SOCS5 was not changed.

## Discussion

SOCS is a large superfamily of proteins that are generally viewed as antagonists of cytokine-induced Janus-activated kinase-STAT signaling. However, SOCS is involved in the regulation of additional signaling pathways, including phosphatidylinositol 3-kinase and ERK MAPK [15]. Studies regarding SOCS and cervical cancer are few in number. SOCS1may play a role in regulating the levels of the E7 protein of human papilloma virus, and affecting their transforming potential [16]. They observed IFN-γ treatment to HeLa and CaSki cell lines resulted in the reduction of E7 protein. Kamio et al. showed overexpression of SOCS1 reduced proliferation rate in both HeLa and CaSki cell lines. They also confirmed SOCS1 suppressed DNA synthesis by observing reduced incorporation of BrdU [16]. However, in the aspect of immune response, SOCS1 silencing enhanced the expression levels of proinflammatory cytokines such as interleukins, TNF-α, IFN-γ resulting in enhanced antigen presentation of dendritic cells [17]. Hence, we suggest the net effect of SOCS1 suppression goes unfavorable for the hosts. Our results also suggest the possibility that SOCS proteins work as tumor suppressors. SOCS1, SOCS3, and SOCS5 expression was lower in cervix cancer than surrounding normal tissue (Fig 2), and the mRNA levels of SOCS genes were lower in cervical cancer cell lines than in normal cervix tissue or fibroblast cell lines (Fig 1). It has previously been reported that marked repression of the transcription of SOCS genes resulted from DNA hypermethylation in the promoter region [6,7,9]. A long list of hypermethylated genes is a common hallmark of all types of human cancer. Although many tumor suppressor genes are silenced by DNA methylation during carcinogenesis, downregulation of SOCS3 and SOCS5 was not dependent on DNA hypermethylation (Figs 3 and 4). Another epigenetic gene silencing mechanism, histone hypoacetylation, mediates SOCS1 downregulation in CaSki, HeLa, and SiHa cells and SOCS3downregulation in CaSKi and SiHa cells (Fig 5). SOCS5 gene expression was not changed at all by the inhibition of DNA methylation or histone acetylation. As Gene silencing often involves a combination of DNA methylation and chromatin histone acetylation, we also examined the combination effect of 5-Aza and TSA on SOCS1 expression in ME-180 cell. However, no significant increase in SOCS1 expression was observed (data not shown). Epigenetic regulation of SOCS genes in cervical cancer cells was summarized in S3 Table. It is worth remembering that we analyzed epigenetic SOCS gene regulation only in four established cervix cancer cell lines and further confirmation is required whether this regulation mechanism is applicable to primary cervix cancer.

Our data showed that altered expression of SOCS1or SOCS3 affected the radiation response of cervical cancer cells. Overexpression of SOCS1 and SOCS3 improved cell survival after ionizing radiation (Fig 6). SOCS1 is an important activator of p53 and the DNA damage response [18]. Because SOCS1 requires the SOCS box to form a complex with ATM, v-Abl- or Pim kinase-mediated phosphorylation could potentially interfere with this interaction and block p53 activation. Therefore, it appears that aberrant phosphorylation by oncogenic kinases could interfere with the tumor suppressor activities of SOCS1 by at least two different mechanisms: (1) phosphorylated SOCS1 would not be able to inhibit the JAK/STAT pathway and to interact with ATM and promote p53 activation [18]. (2) SOCS3improves cell survivalin glioblastoma [11] but acted like a chemosensitizer in anaplastic thyroid cancer [19]. Keratinocyte-specific overexpression of SOCS3 led to atrophied wound-margin epithelia and augmented the inflammatory response of wound keratinocytes by an increase in chemokine (MIP-2) and inflammatory enzyme (COX-2 and iNOS) expression [20]. COX-2 contributes to the metastasis of cervical cancer by promoting autocrine or paracrine VEGF expression at both the primary and metastatic sites [21]. Therefore it is reasonable to suggest that SOCS3 generally acts as a cell survival factor, except in anaplastic thyroid cancer. However, SOCS1 acted like a radiosensitizer in glioblastoma cells [11]. It interrupted the transformation of normal cervical epithelia into neoplastic cells through an E7 protein, like a tumor suppressor. On the other hand, SOCS1 inhibited the apoptosis of islet grafts due to caspase 3 and apoptosis-inducing factor pathways in pancreas of rats [22], indicating that, consistent with our data, it promotes cell survival.

Wild-type cervical cancer cell lines showed repressed expression of SOCS1, SOCS3, and SOCS5. Both DNA methylation and histone deacetylation may contribute to the downregulation of SOCS 1, 3, 5 genes in cervical cancer cells. Recovery of SOCS1 and SOCS3 gene expression decreased the radiosensitivity of HeLa cells. It appears unlikely that the function of SOCS1 and SOCS3 in solid tumors act uniformly in one direction *in vivo*.

## Supporting Information

S1 Table Primer list and PCR condition for MSP. (DOCX)

S2 Table Primer list used for bisulfite sequencing. (DOCX)

S3 Table Summary of epigenetic regulation of SOCS genes in cervical cancer cells. (DOCX)
