# Supplementary material for: Suppressor of Cytokine Signaling (SOCS) Genes Are Silenced by DNA Hypermethylation and Histone Deacetylation and Regulate Response to Radiotherapy in Cervical Cancer Cells
Source: PLoS One. 2015 Apr 7;10(4):e0123133. doi: 10.1371/journal.pone.0123133 (PMC4388447; doi:10.1371/journal.pone.0123133)
Supplement: S1 Table — (DOCX) [file pone.0123133.s001.docx]

**Table S1. Primer list and PCR condition for MSP**

| Name | | Sequence | PCR condition | Ref |
| --- | --- | --- | --- | --- |
| SOCS1 | UmF^a^ | 5'−TGAAGATGGTTTTGGGATTTATGA−3' | 35 cycles of 95°C 30 s  58°C 30 s  72°C 30 s | 7 |
|  | UmR^b^ | 5'−CACAACTCCTACAACAACCACACAC−3' |  |  |
|  | MF^c^ | 5'−TGAAGATGGTTTCGGGATTTACGA−3' |  |  |
|  | MR^d^ | 5'−ACAACTCCTACAACGACCGCACG−3' |  |  |
| SOCS3 | UmF^a^ | 5'−TGTAGATTTTAGTGTATGAAAGTGT−3' | 35 cycles of 95°C 30 s  55°C 30 s  72°C 30 s | this study |
|  | UmR^b^ | 5'−CCAAAAACCAAAAATTAAAAACATA−3' |  |  |
|  | MF^c^ | 5'−TGTAGATTTTAGTGTATGAAAGCGT−3' |  |  |
|  | MR^d^ | 5'−GAAAACCGAAAATTAAAAACGTA−3' |  |  |
| SOCS5 | UmF^a^ | 5'−GAGTTTGTGTTTAGTAGGTTTTTGA−3' | 35 cycles of 95°C 30 s  54°C 30 s  72°C 30 s | this study |
|  | UmR^b^ | 5'−CCTATATAACTACTTCAATCTCCAAT−3' |  |  |
|  | MF^c^ | 5'−GAGTTTGCGTTTAGTAGGTTTTC−3' |  |  |
|  | MR^d^ | 5'−CCTATATAACTACTTCAATCTCCGAT−3' |  |  |

^a^UmF: unmethylated forward, ^b^UmR: unmethylated reverse,

^c^MF: methylated forward, ^d^MR: methylated reverse
