# Supplementary material for: Suppressor of Cytokine Signaling (SOCS) Genes Are Silenced by DNA Hypermethylation and Histone Deacetylation and Regulate Response to Radiotherapy in Cervical Cancer Cells
Source: PLoS One. 2015 Apr 7;10(4):e0123133. doi: 10.1371/journal.pone.0123133 (PMC4388447; doi:10.1371/journal.pone.0123133)
Supplement: S2 Table — (DOCX) [file pone.0123133.s002.docx]

**Table S2. Primer list used for bisulfite sequencing**

| Name | Sequence | Ref |
| --- | --- | --- |
| SOCS1 | 5'−TGTAGGATGGTAGTATATAATTAGGTGGT−3' 5'−TAATACTCCAACAACTCTAAAAAACAATC−3' | 3, 7 |
| SOCS3 | 5'−AGTAGTGATTAAATATTATAAGAAGGT−3'  5'−TCCTTAAAACTAAACCCCCTC−3' | 5, this study |
| SOCS5 | 5'−TTAAAGTTTTGTTTGTTATTGGTTA−3'  5'−ACCTCATCCAAAACACCTAC−3' | this study |
