# Supplementary material for: Suppressor of Cytokine Signaling (SOCS) Genes Are Silenced by DNA Hypermethylation and Histone Deacetylation and Regulate Response to Radiotherapy in Cervical Cancer Cells
Source: PLoS One. 2015 Apr 7;10(4):e0123133. doi: 10.1371/journal.pone.0123133 (PMC4388447; doi:10.1371/journal.pone.0123133)
Supplement: S3 Table — (DOCX) [file pone.0123133.s003.docx]

**Table S3. Summary of epigenetic regulation of SOCS genes in cervical cancer cells**

|  | CaSki | HeLa | ME-180 | SiHa |
| --- | --- | --- | --- | --- |
| SOCS1 | methylation & acetylation | methylation & acetylation acetylation | methylation | acetylation |
| SOCS3 | acetylation | - | - | acetylation |
| SOCS5 | - | - | - | - |
